# Supplementary material for: Analysis of WRKY Gene Family in Acer fabri and Their Expression Patterns Under Cold Stress
Source: Genes (Basel). 2025 Mar 17;16(3):344. doi: 10.3390/genes16030344 (PMC11942518; doi:10.3390/genes16030344)
Supplement: Supplementary file 1 [file genes-16-00344-s001.zip › Table S1.pdf]

Table S1. Basic information of the WRKY gene family in *Acer fabri*

Gene ID	Protein length	relative molecular mass	Theoretical isoelectric point	Instability coefficient	Hydrophilicity hydrophobicity	subcellular localization
Unigene0001032	220	25327.60	8.78	62.74	-0.830	Nucleus
Unigene0001638	359	40084.05	9.81	66.38	-0.843	Nucleus
Unigene0005327	152	17479.85	9.71	45.33	-0.879	Nucleus
Unigene0006082.	409	44913.10	5.74	53.42	-0.793	Nucleus
Unigene0008612	197	21966.80	9.21	67.01	-0.860	Nucleus
Unigene0016335.	55	6496.31	9.64	36.01	-1.347	Nucleus
Unigene0019911	381	42440.40	5.37	55.23	-0.562	Nucleus
Unigene0019967	381	40462.32	8.20	52.65	-0.470	Nucleus
Unigene0021587	364	40308.77	5.39	64.15	-0.967	Nucleus
Unigene0022913	492	53938.23	8.49	56.15	-0.925	Nucleus
Unigene0023495	219	25096.62	7.85	49.08	-0.922	Nucleus
Unigene0024195	1027	111962.45	5.36	52.91	-0.784	Nucleus
Unigene0025263	466	51766.76	8.71	49.87	-0.742	Nucleus
Unigene0026320	586	64671.36	7.74	60.92	-0.992	Nucleus
Unigene0026321	157	17181.88	9.11	50.56	-0.783	Nucleus
Unigene0026322.	406	45138.75	5.69	68.05	-1.073	Nucleus
Unigene0030374	667	73193.29	6.69	56.17	-0.958	Nucleus
Unigene0031204	239	27013.57	6.38	55.87	-1.128	Nucleus
Unigene0036507	293	32247.22	10.04	57.12	-0.791	Nucleus
Unigene0036509	481	53251.90	9.22	45.73	-0.308	Nucleus
Unigene0036753.	534	57846.11	6.17	49.92	-0.668	Nucleus
Unigene0038937	124	14121.44	6.36	52.87	-1.241	Nucleus
Unigene0044579	249	27491.66	8.99	60.73	-0.655	Nucleus
Unigene0045308	55	6506.52	10.33	35.85	-1.433	Nucleus
Unigene0045942	394	42891.57	9.13	50.51	-0.649	Nucleus
Unigene0046253	367	39861.35	5.75	56.76	-0.603	Nucleus
Unigene0047036	395	43318.50	6.07	67.81	-0.829	Nucleus
Unigene0048004	535	58490.46	6.68	48.46	-0.704	Nucleus
Unigene0048493	338	38564.98	5.44	57.40	-0.994	Nucleus
Unigene0050758	314	34339.71	6.56	42.29	-1.025	Nucleus
Unigene0051057	359	40264.37	5.52	47.51	-0.811	Nucleus
Unigene0051368	557	60454.78	7.40	51.00	-0.774	Nucleus
Unigene0052101	266	29857.05	5.00	64.45	-0.917	Nucleus
Unigene0055829	371	41596.01	7.05	52.06	-0.990	Nucleus
Unigene0064835	291	31412.45	9.78	60.56	-0.568	Nucleus
Unigene0067996	124	13753.45	7.25	77.09	-0.685	Nucleus
Unigene0070591	261	29922.98	5.16	60.60	-1.203	Nucleus
Unigene0072283	193	22093.71	9.22	49.39	-1.005	Nucleus
Unigene0074140	124	14398.97	9.55	32.07	-1.303	Nucleus

Unigene0075318	280	31056.46	8.68	46.03	-0.952	Nucleus
Unigene0075858	476	52477.45	8.75	54.48	-0.859	Nucleus
Unigene0075931	126	14295.02	9.59	48.68	-1.023	Nucleus
Unigene0078068	316	35373.56	6.64	63.72	-0.789	Nucleus
Unigene0078383	152	17259.97	4.76	77.00	-0.693	Nucleus
Unigene0079467	326	36313.97	6.13	51.71	-0.944	Nucleus
Unigene0080799	163	17406.85	4.73	55.39	-0.694	Nucleus
Unigene0082468	488	53594.40	5.67	44.75	-0.759	Nucleus
