# Supplementary material for: Analysis of WRKY Gene Family in Acer fabri and Their Expression Patterns Under Cold Stress
Source: Genes (Basel). 2025 Mar 17;16(3):344. doi: 10.3390/genes16030344 (PMC11942518; doi:10.3390/genes16030344)
Supplement: Supplementary file 1 [file genes-16-00344-s001.zip › Table S2.pdf]

Table S2. qRT-PCR Primer

Number	• Gene name	Primer sequence
1	Unigene0026320	F: GGTTAGTTCTAATGTAATTCAGGGGT
		R: GGATTTACTCCTCTGCGATGC
2	Unigene0026321	F: GCTATGACCAATCTTTCCCTCC
		R GGTATGCCTTCCGAATCCTG
3	Unigene0026322	F: TTCTTCAACCGTTGTTACAGTGG
		R: GGTAGTATAGTGCGATGATTGCG
4	Unigene0036509	F: GCTCCTGTAAATCCCCTCCC
		R: CCTTAAAAGCAGATACTTGTTCGTT
5	Unigene0036753	F: ACTGGGGTCCAAACAAGGC
		R: GAGCACGAACCGAAACACG
6	Unigene0047036	F: GGTTCTGGTTCATCTTTTGGGT
		R: CGGTGGTGTAAAAAACGGCT
7	Unigene0048493	F: ATTGAAAAATGGAGGCGGC
		R: TGAATCTTCGGACTTGCGG
8	Unigene0064835	F: GGCTTGTTTCATCCAGTCGC
		R: CCTCCTTGCTTTCCGTTCG
9	Unigene0075318	F: AGAGGGTTAGTGAAGAAAACAGGAA
		R: AGTGGAACTGCTATCGGAGACAT
10	Unigene0078068	F: TTGTTTGATATGGTAAAGCCGC
		R: TGACAGCCTCACTTGACGCA
